# Social Contacts and Transmission of COVID-19 in British Columbia, Canada

**DOI:** 10.3389/fpubh.2022.867425

**Published:** 2022-05-03

**Authors:** Notice Ringa, Sarafa A. Iyaniwura, Samara David, Mike A. Irvine, Prince Adu, Michelle Spencer, Naveed Z. Janjua, Michael C. Otterstatter

**Affiliations:** ^1^Data and Analytic Services, British Columbia Centre for Disease Control, Vancouver, BC, Canada; ^2^School of Population and Public Health, University of British Columbia, Vancouver, BC, Canada; ^3^Department of Mathematics, Institute of Applied Mathematics, University of British Columbia, Vancouver, BC, Canada; ^4^Faculty of Health Sciences, Simon Fraser University, Burnaby, BC, Canada

**Keywords:** social contacts, COVID-19, transmission control, correlation, regression

## Abstract

**Background:**

Close-contact rates are thought to be a driving force behind the transmission of many infectious respiratory diseases. Yet, contact rates and their relation to transmission and the impact of control measures, are seldom quantified. We quantify the response of contact rates, reported cases and transmission of COVID-19, to public health contact-restriction orders, and examine the associations among these three variables in the province of British Columbia, Canada.

**Methods:**

We derived time series data for contact rates, daily cases and transmission of COVID-19 from a social contacts survey, reported case counts and by fitting a transmission model to reported cases, respectively. We used segmented regression to investigate impacts of public health orders; Pearson correlation to determine associations between contact rates and transmission; and vector autoregressive modeling to quantify lagged associations between contacts rates, daily cases, and transmission.

**Results:**

Declines in contact rates and transmission occurred concurrently with the announcement of public health orders, whereas declines in cases showed a reporting delay of about 2 weeks. Contact rates were a significant driver of COVID-19 and explained roughly 19 and 20% of the variation in new cases and transmission, respectively. Interestingly, increases in COVID-19 transmission and cases were followed by reduced contact rates: overall, daily cases explained about 10% of the variation in subsequent contact rates.

**Conclusion:**

We showed that close-contact rates were a significant time-series driver of transmission and ultimately of reported cases of COVID-19 in British Columbia, Canada and that they varied in response to public health orders. Our results also suggest possible behavioral feedback, by which increased reported cases lead to reduced subsequent contact rates. Our findings help to explain and validate the commonly assumed, but rarely measured, response of close contact rates to public health guidelines and their impact on the dynamics of infectious diseases.

## Introduction

A wide variety of infectious respiratory diseases, including influenza, measles, plague, tuberculosis and the new and ongoing Coronavirus Disease 2019 (COVID-19), are transmitted largely through close-contact and spread based on the social contacts and mixing patterns of the host population (1–3). Effective contacts (interactions that allow pathogen transfer between individuals) typically involve inhalation of infectious secretions from coughing, sneezing, laughing, singing or talking, but may also include touching contaminated body parts or surfaces followed by ingestion of the pathogen (4). Control strategies against such infections are based on contact avoidance measures, including isolation of those who are ill, use of personal protective equipment such as gloves and face masks, and physical distancing (5, 6). In this study, we examine the relations between self-reported social contact patterns, public health control measures, and the dynamics of COVID-19 in the province of British Columbia (BC), Canada. The history and epidemiological features of COVID-19 have been documented by several studies including in (7–14), and we present a summary of these as well as conventional COVID-19 transmission control measures in Appendix 1.

A small number of studies, including in (15–18), have analyzed population patterns of social contacts, and their connection to the dynamics of close-contact infectious diseases. Overall, the studies show that disease incidence and effective reproduction number (average number of newly infected individuals per case) increase with contact rates. However, contact rates and their effects on infection dynamics may vary over time and with factors such as geographical location, sex, age, household size, occupation and other socio-economic factors.

In our study, we explore and quantify associations between social contact patterns, public health orders, transmission, and reported cases of COVID-19, in BC and in the two most populous BC regional health authorities: Fraser Health Authority (FHA) and Vancouver Coastal Health Authority (VCHA) (19). We make use of detailed contact survey data and estimate transmission using a model-based metric of the time-varying reproductive number, *Rt*. We specifically consider data from autumn of 2020 onward, during which a series of regional and provincial public health orders were introduced to reduce the number of close contacts and curb transmission.

## Methods

We studied the association between close-contact rates [based on the BC Mix COVID-19 Survey data, which is summarized in Appendix 2 and described in detail in (20)], daily new confirmed COVID-19 cases [obtained from BC COVID-19 data, which is provided by the BC Centre for Disease Control (21), and also available at (22)] and *R*_*t*_ [derived by fitting the *covidseir* transmission model of (7), where *R*_*t*_ was computed using the Next-Generation matrix method (23, 24), to the reported case data] in BC, from September 13, 2020 to February 19, 2021, a period in which three public health contact-restriction orders were introduced (October 26, November 7 and November 19). Further details of the public health orders are provided in Appendix 3. For each successive four-day period, we calculated (i) population rates of contact as the average number of self-reported close-contacts made by an individual in a day (average daily contacts); (ii) the average number of newly reported COVID-19 cases per day (average daily cases or new cases); and (iii) transmission rate of COVID-19 as the average daily value of our model-based estimate of *R*_*t*_. We used segmented linear regression [described in Appendix 4 and (25–27)] to investigate the impact of public health orders on the three variables. We used Pearson correlation [summarized in Appendix 5 and described in detail in (28–31)] to assess the instantaneous relationship between contact rates and *R*_*t*_. Finally, we used vector autoregressive (VAR) models [described in Appendix 6 and in (32–35)] to quantify lagged associations between contact rates, new cases and *R*_*t*_. All analysis was performed using R version 3.6.3. We use α = 0.05 for all statistical tests.

## Results

### Effects of Public Health Orders on Average Daily Contacts, Average Daily Cases and Transmission

**Figure 1:**
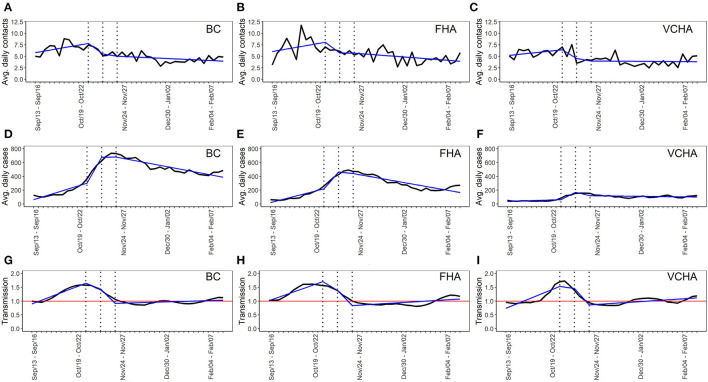
Time series of average daily contacts (contact rates), average daily cases (new cases) and transmission (R_t_) of COVID-19 in BC **(A,D,G)**, FHA **(B,E,H)** and VCHA **(C,F,I)** from September 13, 2020 to February 19, 2021. The vertical dotted lines indicate dates of announcement of public health contact-restriction orders on October 26, 2020, November 07, 2020 and November 19, 2020. Each plot contains derived segmented linear regression lines with three knots at the dates of introduction of the public health orders. Horizontal lines in the plots for transmission indicate the transmission threshold R_t_ = 1.

Provincially, rising contact rates and transmission (*R*_*t*_) reversed shortly after the first health order on October 26, 2020 (Figures 1A,G); for contacts, this declining trend lasted only until the second public health order (13 days later, on November 7), whereas for *R*_*t*_, the decline continued to at least the third order (25 days later, on November 19).

Both contact rates and *R*_*t*_ were relatively stable after the third order until the end of our study period (February 19, 2021). As expected, the trend in new cases mirrored that of our transmission indicator but was shifted about 2 weeks later, corresponding to the delay between transmission to symptom onset followed by diagnosis, and case reporting (Figures 1D–G). The same patterns were generally apparent in both of the regional health authorities we studied, although declines in contact rates and *R*_*t*_ appeared to start roughly 1 week before the first public health order in FHA, and roughly 1 week after the first order in VCHA (Figures 1B–I). Simple comparison of overall contact rates and *R*_*t*_ before and after the introduction of public health orders indicated that in BC, FHA and VCHA, contact rates declined by 30.1, 29.2, and 29.9%, while *R*_*t*_ declined by 17.9, 25.0, and 5.4%, respectively, following the first public health order onwards.

Our segmented linear regression models showed that in BC, FHA and VCHA, the slope of the contact rate regression line was positive before the first public health order, turned substantially negative thereafter and slightly increased, but remained negative or close to zero through all other health orders (Table 1).

**Table 1:** Slopes of regression lines of average daily contacts and transmission in the province and in FHA and VCHA, within the four time intervals separated by the three dates (Π_1_, Π_2_ and Π_3_) of announcement of public health orders, based on associated model estimates β_1_, β_2_, β_3_ and β_4_ presented in Supplementary Tables S3, S5 in Appendix 4.

	***t*** **≤ Π_1_**	**Π_1_ ≤*t* ≤ Π_2_**	**Π_2_ ≤*t* ≤ Π_3_**	***t*** **≥ Π_3_**
Slope of BC average daily contacts	0.184^**^	−0.768^***^	−0.159	−0.048
Slope of FHA average daily contacts	0.185	−0.779^*^	−0.013	−0.079
Slope of VCHA average daily contacts	0.111	−0.634^**^	−0.182	−0.007
Slope of BC transmission	0.068^***^	−0.071^***^	−0.173^***^	0.005^***^
Slope of FHA transmission	0.063^***^	−0.105^***^	−0.184	0.011^***^
Slope of VCHA transmission	0.072^***^	−0.025^***^	−0.199^***^	0.011^***^

* *p < 0.1;*

** *p < 0.05;*

*** *p < 0.01*.

The changes in contact rate slope after the first public health order (i.e., Π_1_ ≤ *t* ≤ Π_2_) were statistically significant in the province and in VCHA (*p* < 0.05), but not in FHA. Provincially and in the two regional health authorities, the changes in contact rate slope following the second and the third health orders (i.e., Π_2_ ≤ *t* ≤ Π_3_ and *t* ≥ Π_3_) were not statistically significant (*p* > 0.05). Provincially and in the two regional health authorities, the slope for transmission (*R*_*t*_) was positive before the first public health order, turned negative after this order, decreased further following the second public health order, and stabilized after the third health order (Table 1). Changes in transmission slope following all public health orders were statistically significant (*p* < 0.05), except after the second health order in FHA.

### Pearson Correlation of Average Daily Contacts and Transmission

Our correlation analysis showed that high contact rates and high transmission tended to occur at the same time. Provincially, and in both regional health authorities, transmission (average daily *R*_*t*_) was significantly positively correlated with average daily contacts (*r*^*BC*^ = 0.64, p < 0.001); *r*^*FHA*^ = 0.53, p < 0.001; *r*^*VCHA*^ = 0.34, *p* = 0.033). Based on these values, the magnitude of the correlation was about 50% stronger in FHA compared to VCHA (*r*^*FHA*^ = 1.56 × ^*VCHA*^).

### VAR Models of Average Daily Contacts and Average Daily Cases, and Average Daily Contacts and Transmission

**Figure 2:**
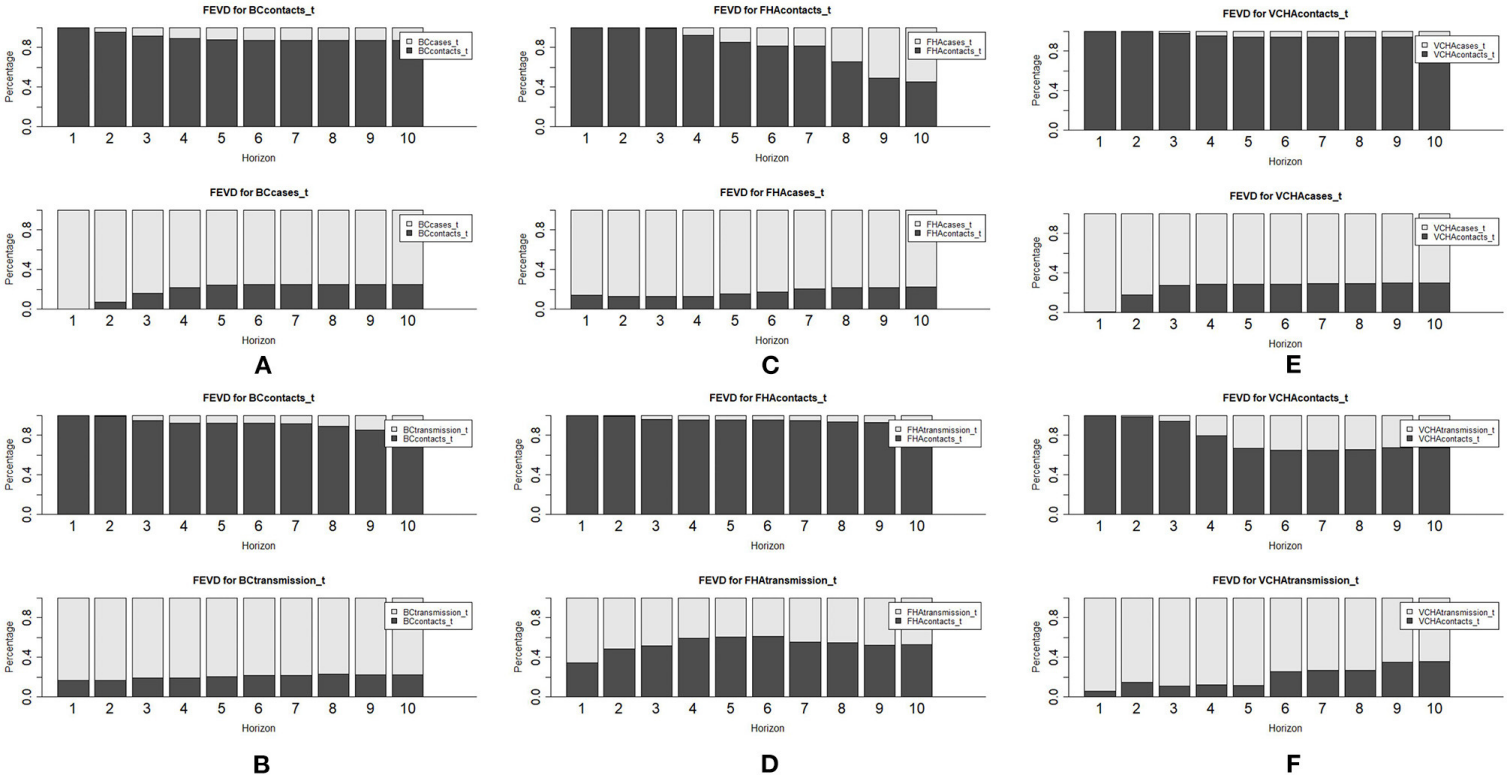
Forecast error variance decomposition (FEVD) results for VAR models of average daily contacts and cases and average daily contacts and transmission in BC **(A,B)**, FHA **(C,D)**, and VCHA **(E,F)**.

The notations *BC*__*contacts*__t_, *BC*_*casest*_ and *BC*__*transmission*__t_ represent the (stationary) time series of average daily contacts, cases, and transmission, respectively, in BC. The corresponding notations for FHA and VCHA are similarly defined. Our time series models showed that variation in new cases and transmission of COVID-19 were significantly attributable to past values of average daily contacts, whereas variation in average daily contacts was explained largely by its own past values (Figure 2).

Each panel of the FEVD plots shown in Figure 2 illustrates the proportion of variation in cases, contacts or transmission that is explained by that variable's own past values vs. the past values of other variables.

Provincially, on average, about 19% of the variation in average daily cases, and about 20% of the variation in COVID-19 transmission, was explained by previous rates of daily contact (Figures 2A,B). In FHA, previous average daily contacts contributed up to 22% of the variation in average daily cases (Figure 2C) and up to 61% of the variation in transmission (Figure 2D). In VCHA, up to 30% of the variation in average daily cases was explained by average daily contacts, whereas contact rates explained up to 36% of the variation in transmission (Figures 2E,F). Supplementary Table S13 in Appendix 6.5 shows numerical representations of all FEVD plots in Figure 2.

Granger causality testing confirmed that provincially and for VCHA, previous daily contacts were a significant time series driver of average daily cases (BC: *p* = 0.006, VCHA: *p* = 0.011), but the same did not hold for FHA (see Table 2). Supplementary Figure S4 in Appendix 6.5 provides a visual description of the Granger causality testing results in Table 2.

**Table 2:** Granger causality test results for average daily contacts and average daily cases and average daily contacts and transmission, in BC and two health regions, FHA and VCHA.

*BC*__*contacts*__t_ G-causes *BC_*case*_*s*__t__* (*p* = 0.006)	*BC*__*contacts*__t_ does not G-cause *BC_*transmissio*_*n*__t__* (*p* = 0.945)
*BC_*case*_*s*__t__* G-causes *BC*__*contacts*__t_ (*p* = 0.049)	*BC_*transmissio*_*n*__t__* does not G-cause *BC*__*contacts*__t_ (*p* = 0.544)
*FHA*__*contacts*__t_ does not G-cause *FHA_*case*_*s*__t__* (*p* = 0.519)	*FHA*__*contacts*__t_ does not G-cause *FHA_*transmissio*_*n*__t__* (*p* = 0.574)
*FHA_*case*_*s*__t__* G-causes *FHA*__*contacts*__t_ (*p* = 0.001)	*FHA_*transmissio*_*n*__t__* does not G-cause *FHA*__*contacts*__t_ (*p* = 0.582)
*VCHA*__*contacts*__t_ G-causes *VCHA_*case*_*s*__t__* (*p* = 0.011)	*VCHA*__*contacts*__t_ G-causes *VCHA_*transmissio*_*n*__t__* (*p* = 0.017)
*VCHA_*case*_*s*__t__* does not G-cause *VCHA*__*contacts*__t_ (*p* = 0.537)	*VCHA_*transmissio*_*n*__t__* G-causes *VCHA*__*contacts*__t_ (*p* = 0.023)

Our time series models also showed that some variation in average daily contacts was explained by previous average daily cases and transmission of COVID-19. Provincially, average daily cases and transmission explained up to 13% (or 10% on average) and up to 18%, respectively, of the variation in average daily contacts (Figures 2A,B). In FHA, past average daily cases contributed up to 55% of the variation in the contact rates (Figure 2C), whereas previous transmission rates contributed up to 7% to the variation in average daily contacts in (Figure 2D). In VCHA, the reverse was true with previous average daily cases explaining little (up to 6%) variation in average daily contacts, but transmission explaining up to 35% of the variation in average daily contacts (Figures 2E,F).

The impact of previous case counts on average daily contacts was significant at the provincial level and in FHA (BC: *p* = 0.049; FHA: *p* = 0.001), but not significant for VCHA. Past values of average daily contacts did not significantly impact transmission provincially or in FHA; however, these two variables were significantly associated in VCHA.

## Discussion

The primary approach to prevent the spread of many infectious diseases transmissible through close person-to-person contact is reduction or avoidance of such contacts altogether. Yet, few studies have quantified the impact that such contact-restrictions have on rates of “effective” contact (those actually involved in transmission) and on transmission itself. In our study, we explored time series relationships between close contact patterns and the dynamics of the ongoing COVID-19 pandemic in British Columbia, Canada and in its two most populous regional health authorities, FHA and VCHA, from mid-September, 2020 to mid-February, 2021. During this period, three public health contact-restriction measures were introduced (on October 26, November 7 and November 19) to control rising numbers of cases. We used data from the BC Mix Survey, which specifically captures rates of close contacts that are likely to underlie transmission. We analyzed contact rates in relation to the timing of contact-restriction measures and assessed their impact on COVID-19 transmission (average daily number of new infections generated per case, *R*_*t*_) and reported new cases.

We found that in BC, FHA and VCHA, all three public health orders reduced contact rates and transmission, or helped to maintain lowered rates. Overall, declines in contact rates and transmission occurred concurrently with the announcement of public health orders, whereas declines in newly reported cases were, as expected due to reporting delays, lagged by roughly 2 weeks. The decline we observed in contact rates in FHA about 1 week prior to the public health orders could have resulted from public anticipation and early media reporting of the upcoming restriction orders and/or from reports of rising numbers of new cases of COVID-19. Contact rates declined by roughly 30% overall after the first public health order. Transmission similarly declined in response to these orders, although this effect varied by region (*R*_*t*_ reduced by 17.9, 25.0, and 5.40% in BC, FHA and VCHA, respectively). This observation suggests that compliance to public health orders by limiting the frequency of person-to-person contacts played an important role in reducing the transmission of COVID-19. In all regions, transmission curves mirrored, and were highly correlated with those of contact rates, suggesting that these self-reported rates of close contact were directly and concurrently related to spread of COVID-19. Through time series analysis, we showed that lagged daily contacts significantly predicted, and explained roughly 19% of the variation in subsequent new cases at the provincial level. Interestingly, we also found evidence of behavioral feedback at the population level, whereby increased reported cases led to reduced subsequent rates of contact: overall, previous daily cases explained about 10% of the variation in subsequent daily contacts in the province. The interdependence of previous contact rates, new cases and transmission of COVID-19 varied by region.

It is important to note that our time series analysis only assesses the impact of previous or lagged contacts on transmission and new cases, i.e., it does not include the impact of concurrent contacts. Hence, we find that previous contacts primarily impact numbers of new cases, where there is naturally a delay due to reporting, rather than rates of transmission (where the impact is expected to largely occur concurrently). However, we show through our correlation analysis that contacts and transmission are significantly concurrently related.

A few studies have quantified variation in transmission or cases of an infectious disease as a function of contact rates. For instance, in (16), the authors analyzed United Kingdom contact survey data during periods before and after the March 2020 lockdown due to the COVID-19 pandemic, and found that a model-derived effective reproduction number declined by 75% as a response to a 74% reduction in average daily contacts. In (15), the authors studied contact survey data from Belgium during different stages of intervention against COVID-19 and found that an 80% decline in the average number of contacts during the first lockdown period resulted in a decline of the effective reproduction number to below one, resulting in fewer reported new cases. In (36), the authors studied United Kingdom population mixing patterns during the 2009 H1N1 virus influenza epidemic and found that a 40% reduction in contacts among school children during school holidays resulted in about 35% decline in the reproduction number of influenza. These studies confirm a relation between self-reported contact rates and infectious disease transmission, but also show variation that may be due to epidemiological factors such as difference in the transmission environment (e.g., use of personal protective equipment) and the types of contacts being measured. Other studies that have explored the control of COVID-19 by management of social contacts include (37, 38), which indicated that the relatively low transmission rate of COVID-19 in India in early 2020, was attributable to public compliance to a strict government-imposed lockdown on social gatherings.

The possibility of a feedback mechanism in which contacts rates decrease as a result of increasing transmission and new cases, has been documented in some previous studies. For instance, during the 2014 Ebola outbreak in Sierra Leone, self-reported prevention practices such as avoidance of contacts with corpses, were found to have increased with rising disease prevalence (39). During the early stages of the COVID-19 pandemic, the practice of cautious social contacts by the Singaporean population, increased with rising rates of infection due to behavioral drivers such as fear and perceived risk of infection (40). Similarly, the decline of close contacts in Hong Kong during the first quarter of 2020 is thought to have resulted from increasing messaging and spread of information about the prevalence of COVID-19 (41). Thus, wide-spread public awareness of increasing numbers of new cases, through public health and various information media, may help to explain population reductions in contact rates.

In our study, we found that contact patterns and the related dynamics of COVID-19 varied with the geographies considered. A number of previous studies have also identified variation in contact rates by geography, and by factors that themselves vary geographically. In (17), the authors analyzed and compared social contact survey data for eight European countries in 2005 and 2006, and found that contact rates varied by geographical location, but also by sex, age and household size. In (42), the authors reviewed contact survey data across several countries from varying economic brackets and found that, in general, high contact rates were associated with densely populated settings and large household sizes, which characterized most low to middle-income countries. This is consistent with the general expectation that close-contact infectious diseases are more likely to impact densely populated regions and settings with large household sizes. Geographic variation in our results, particularly the higher contact rates, transmission and numbers of new cases in FHA compared to VCHA, may reflect the generally higher population density and larger household sizes in FHA (19). Related to the above factor is the evidence that the geographic spread of COVID-19 cases is connected to the local economic structure of a location relative to neighboring regions–in Italy, COVID-19 hit economic core locations (which were also characterized by higher populations densities) harder than regions with lower economic activities (43). Variations in close contact, case counts and transmission of COVID-19 can offer guidance for shaping or relaxing public health restrictions (44). For instance, a more rapid deployment of control measures can be applied in densely populated regions reporting high contact rates and cases than in sparsely distributed populations; and control measures can be tailored to capture population heterogeneity and other infection risk factors such as age groups.

Our analysis has several important limitations. We relied on case surveillance data to determine the number of new cases and the transmission indicator of COVID-19 over time. This means we did not account for asymptomatic infection, which may be a strong driver of COVID-19 transmission, and could have impacted the conclusions of our study. Relying on case surveillance data may also underestimate the actual number of new cases in settings where symptomatic individuals did not seek testing or where testing capacity is constrained by inaccessibility or shortage of resources. Three regional health authorities were not included in the assessment of regional associations of contact rates to COVID-19 dynamics - the Northern, Interior and Vancouver Island Health Authorities. These health authorities have relatively smaller population sizes, are more sparsely populated and have many rural communities (19). In these health authorities, self-reported contact rate data were too sparse for us to explore relations with reported cases and transmission. As a result, this study may not be representative of patterns in more rural populations. Limitations of the self-reported contact rates that may affect our analysis are provided in (20). For instance, some population groups including the economically marginalized, the under-housed, and those in immigration detention or incarceration, are likely underrepresented in the survey. In this study, we compared time series of means (averages) of daily contacts, cases and transmission of COVID-19, and did not consider other measures of central tendency, which may be crucial when analyzing skewed data. For instance, in the early stages of the COVID-19 pandemic contact rates were possibly higher during social gatherings over holidays, while more cases of COVID-19 tended to be reported on days after weekends and on days following holidays (45). Our conclusions may also be impacted by the choice of the time series analysis methods employed-in (46), the authors showed how the choice of the best times series analysis method can depend on factors such as the stage of an outbreak and the granularity of the geographic level explored.

This is the first study analyzing extensive and novel data on person-to-person contacts collected continuously throughout the province of British Columbia, Canada to understand the role of close contacts in transmission and control of infectious diseases. The study provides a quantitative approach to measuring the temporal associations among self-reported close contact rates, public health contact-restriction orders, and transmission dynamics of COVID-19. The observed impacts of person-to-person contacts on COVID-19 dynamics, as well as the capability of public health measures to modify these contact rates, are likely to prevail, although with varying magnitudes, in other jurisdictions and for other infectious diseases with similar modes of transmission. These findings support the quantitative study of population contact rates, which can inform infectious disease control strategies.

## Data Availability Statement

The raw COVID-19 case data used in this article was extracted from a line list generated by BCCDC Public Health Reporting Data Warehouse (PHRDW). The contact rate data used in this study was retrieved from the BC Mix COVID-19 survey and may be available upon reasonable request.

## Ethics Statement

The study was approved by the University of British Columbia Behavioral Research Ethics Board (No: H20-01785).

## Author Contributions

NR and MO developed this concept along with NJ. All authors reviewed and agreed on the final submission.

## Funding

This study was supported by Canadian Institutes of Health Research (CIHR Grant No. VR5-172683).

## Conflict of Interest

The authors declare that the research was conducted in the absence of any commercial or financial relationships that could be construed as a potential conflict of interest.

## Publisher's Note

All claims expressed in this article are solely those of the authors and do not necessarily represent those of their affiliated organizations, or those of the publisher, the editors and the reviewers. Any product that may be evaluated in this article, or claim that may be made by its manufacturer, is not guaranteed or endorsed by the publisher.
