# Supplementary material for: Social Contacts and Transmission of COVID-19 in British Columbia, Canada
Source: Front Public Health. 2022 May 3;10:867425. doi: 10.3389/fpubh.2022.867425 (PMC9110764; doi:10.3389/fpubh.2022.867425)

Appendices

Appendix 1: COVID-19 epidemiology and control measures

COVID-19, a viral respiratory infectious disease caused by Severe Acute Respiratory Syndrome Coronavirus 2 (SARS-CoV-2), emerged in Wuhan, China in 2019 and has since spread worldwide to over 270 million people and led to over 5.3 million deaths [7, 8, 9, 10, 11, 12, 13, 14]. The primary mode of transmission for COVID-19 is contact with and inhalation of infectious respiratory droplets, which may propel two meters or more before settling on environmental surfaces. Symptoms of COVID-19 include fever or chills, cough, shortness of breath, fatigue, muscle or body aches, headache, loss of taste or smell, sore throat, which may result in mild to severe illness, and death. Governments around the world have attempted to contain the spread of COVID-19 by restricting social gatherings, closing schools, suspending recreational activities and travel, and by other means of reducing contacts. Nevertheless, the impacts of public health measures on actual rates of close contact, and the relation between these contacts and transmission, are rarely quantified.

Appendix 2: Description of BC Mix COVID-19 Survey contact rate data

BC Mix COVID-19 Survey is an ongoing online survey launched by the British Columbia Centre for Disease Control (BCCDC) on September 04, 2020. These survey data are used to measure and assess contact patterns and activity levels in BC during the COVID-19 pandemic and to inform the timing of easing and re-imposing physical distancing measures. The survey comprises 94 items across six key domains, namely demographic information; COVID-19 testing and results, symptoms, and health behaviors; activities and behavior in and outside of the home; internet and social media use; perceptions and attitudes around COVID-19; and COVID-19 vaccine acceptance. Survey participants are aged 18 years and above. A detailed description of the survey including its development, design, case definitions and other characteristics is described in [20]. Survey respondents record numbers of close contacts made in a single day, in answer to the question " How many people did you have in-person contact with between 5 am yesterday and 5 am today?". In the survey, in-person contact is defined as face-to-face two-way conversation with three or more words, or physical skin-to-skin contact such as a handshake, hug, kiss and contact sports. For the purposes of this study, we used weighted survey data including only the "baseline" responses (i.e., participants can complete the survey multiple times, but we included only the first completed survey for each individual). Weighting was used to correct for differences between the distribution of respondents (by age, sex, geography and ethnicity) and that of the BC population.

Appendix 3: Public health contact-restriction orders in British Columbia

The first case of COVID-19 in BC was detected on January 28, 2020, after which rapid spread of the virus led to a number of public health contact-restriction orders [22]. At various times, these orders included temporary reduction in the sizes of social gatherings, avoidance of non-essential travel, strict physical distancing, cancellation of sporting activities, shut-down of businesses, school closures, work-from-home arrangements, mandatory indoor masking and quarantine for

travelers. These orders were associated with marked declines in transmission after the initial peak in spring of 2020 (the so-called ’first wave’), with only 30-40 new cases reported daily in BC throughout the summer of 2020. Nevertheless, cases grew rapidly during autumn of 2020 (the ’second wave’): during November alone, the number of newly reported cases quadrupled, from roughly 200 per day to 800 cases per day. On October 26, 2020, the province restricted social gatherings to household members plus their immediate six close-contacts; on November 07, 2020, additional restrictions limited social gatherings to household members only for the two most populous regional health authorities (FHA and VCHA); and on November 19, 2020, mandatory indoor masking was announced, and social gatherings were limited to household members only for the entire province. These restrictions were followed by a steady decline in newly reported COVID-19 cases during December 2020.

Appendix 4: Segmented linear regression

The general linear segmented regression model of a dependent variable v and independent variable t with k knots $\Pi_1, ..., \Pi_k$, is given by

$$v = \beta_0 + \beta_1 t + \beta_2(t - \Pi_1)_+ + \beta_3(t - \Pi_2)_+ + \ldots + \beta_{k+1}(t - \Pi_k)_+, \qquad (1)$$

where β_0, β_1, β_2,..,β_{k+1} are constants to be determined, and the terms $(u)_+$ have the value u if u is positive, and 0 otherwise [25,26,27]. Equation (1) can also be written as

$$v = \begin{cases} \beta_0 + \beta_1 t, & t \le \Pi_1 \\ \beta_0 + (\beta_1 + \beta_2)t - \beta_2\Pi_1, & \Pi_1 \le t \le \Pi_2 \\ \vdots & \vdots \\ \beta_0 + (\beta_1 + \beta_2 + \ldots + \beta_{k+1})t - \beta_2\Pi_1 - \beta_3\Pi_2 - \ldots - \beta_{k+1}\Pi_k, & t \ge \Pi_k. \end{cases} \qquad (2)$$

Thus, the v-intercept and slope of the first segment are β_0 and β_1, respectively; the intercept and slope of the second segment are $\beta_0 - \beta_2\Pi_1$ and $\beta_1 + \beta_2$, respectively; and the intercept and slope of the $k + 1^{th}$ segment are $\beta_0 - \beta_2\Pi_1 - \beta_3\Pi_2 - ... - \beta_{k+1}\Pi_k$ and $\beta_1 + \beta_2 + ... + \beta_{k+1}$, respectively. The equations of the regression lines for our time series of contact rates, new cases and transmission take the form

$$v = \begin{cases} \beta_0 + \beta_1 t, & t \leq \Pi_1 \\ \beta_0 + (\beta_1 + \beta_2)t - \beta_2\Pi_1, & \Pi_1 \leq t \leq \Pi_2 \\ \beta_0 + (\beta_1 + \beta_2 + \beta_3)t - \beta_2\Pi_1 - \beta_3\Pi_2, & \Pi_2 \leq t \leq \Pi_3 \\ \beta_0 + (\beta_1 + \beta_2 + \beta_3 + \beta_4)t - \beta_2\Pi_1 - \beta_3\Pi_2 - \beta_4\Pi_3, & t \geq \Pi_3, \end{cases} \quad (3)$$

where $v = v(t)$ is the regression-estimated value of a variable v at time t; Π_1, Π_2 and Π_3 are the dates of announcement of the first, second and third public health order, respectively; β_0, β_1, β_2, β_3 and β_4 are constants to be estimated based on time series data. We fit segmented linear regression models to our time series and generated estimates (i.e. β_0, β_1, β_2, β_3 and β_4) for each regression line using the *segmented* R-package.

According to Equation (3), for each regression line in Figure 1, the slope of the first, second, third and fourth segment is given by β_1, $\beta_1 + \beta_2$, $\beta_1 + \beta_2 + \beta_3$ and $\beta_1 + \beta_2 + \beta_3 + \beta_4$, respectively. Thus, β_2 represents the change in the regression slope from the interval $t \leq \Pi_1$ to $\Pi_1 \leq t \leq \Pi_2$; β_3 represents the change in the regression slope from the interval $\Pi_1 \leq t \leq \Pi_2$ to $\Pi_2 \leq t \leq \Pi_3$ and β_4 represents the change in the regression slope from the interval $\Pi_2 \leq t \leq \Pi_3$ to $t \geq \Pi_3$.

Appendix 4.1: Estimates for linear segmented regression lines in Figure 1

This section presents tables of estimates (i.e. β_0, β_1,..., β_4) for linear segmented regression lines in Figure 1, where the breakpoints, Π_1, Π_2 and Π_3, are located at the dates of the three BC public health orders on October 26, 2020, November 07, 2020 and November 19, 2020. The number of '*'s besides each estimate increases with the statistical significance of the estimate, and the values inside brackets () are standard errors for the approximation of the estimates.

Table 3: Estimates for segmented linear regression lines for average daily contacts in BC, FHA and VCHA, shown in **Figures 1(A)**, **1(B)** and **1(C)**, respectively.

Parameter	***Regression estimates for average daily contacts***		
	BC	FHA	VCHA
β_1	0.184** (0.073)	0.185 (0.126)	0.111 (0.080)
β_2	−0.952*** (0.330)	−0.964*(0.568)	−0.745** (0.361)
β_3	0.609 (0.506)	0.766 (0.870)	0.452 0.553)
β_4	0.111 (0.272)	−0.066 (0.467)	0.175 (0.297)
β_0	5.599*** (0.555)	5.834*** (0.956)	5.085*** (0.608)
Observations	40	40	40
R^2	0.643	0.384	0.482
Adjusted R^2	0.603	0.313	0.423

Residual Std. Error	0.916 (df = 35)	1.576 (df = 35)	1.002 (df = 35)
F Statistic	15.791*** (df = 4; 35)	5.451*** (df = 4; 35)	8.149*** (df = 4; 35)

Note: *p<0.1; **p<0.05; ***p<0.01

Table 4: Estimates for segmented linear regression lines for average daily cases in BC, FHA and VCHA, shown **in Figures 1(D)**, **1(E)** and **1(F)**, respectively.

Parameter	***Regression estimates for average daily cases***		
	BC	FHA	VCHA
β_1	21.863*** (3.528)	18.159*** (3.353)	2.436* (1.244)
β_2	103.238*** (15.888)	63.257*** (15.099)	32.876*** (5.602)
β_3	−123.977***(24.347)	−89.144*** (23.139)	−51.862*** (8.585)
β_4	−14.387 (13.086)	−4.763 (12.436)	15.696*** (4.614)
β_0	39.411 (26.750)	1.884 (25.423)	33.603*** (9.433)
Observations	40	40	40
R^2	0.955	0.909	0.836
Adjusted R^2	0.950	0.899	0.818
Residual Std. Error	44.125 (df = 35)	41.936 (df = 35)	15.560 (df = 35)
F Statistic	184.430*** (df = 4; 35)	87.611*** (df = 4; 35)	44.761*** (df = 4; 35)

Note: *p<0.1; **p<0.05; ***p<0.01

Table 5: Estimates for segmented linear regression lines for transmission indicator R_t for BC, FHA and VCHA, shown in **Figures 1(G), 1(H)** and **1(I)**, respectively.

Parameter	***Regression estimates for transmission indicator R_t***		
	BC	FHA	VCHA
β_1	0.068*** (0.005)	0.063*** (0.008)	0.072*** (0.009)
β_2	−0.139*** (0.024)	−0.168*** (0.036)	−0.097** (0.041)
β_3	−0.102*** (0.037)	−0.079 (0.055)	−0.174*** (0.063)
β_4	0.178*** (0.020)	0.195*** (0.029)	0.210*** (0.034)
β_0	0.829*** (0.041)	0.951*** (0.060)	0.664*** (0.069)
Observations	40	40	40
R^2	0.925	0.877	0.782
Adjusted R^2	0.916	0.862	0.757
Residual Std. Error	0.067 (df = 35)	0.099 (df = 35)	0.114 (df = 35)
F Statistic	107.818*** (df = 4; 35)	62.113*** (df = 4; 35)	31.455*** (df = 4; 35)

Note: *p<0.1; **p<0.05; ***p<0.01

Appendix 5: Pearson correlation

Correlation analysis is a statistical method for determining the strength of association between two variables. The relationship between the variables is defined by a correlation coefficient r, which varies between -1 and +1, such that -1 indicates strong negative association while +1 indicates strong positive correlation between the variables [28, 29, 30, 31]. The correlation coefficient of Person correlation of two variables x and z is given by

$$r = \frac{\sum(x - m_x)(z - m_z)}{\sqrt{\sum(x - m_x)^2 (x - m_z)^2}} \qquad (4)$$

where m_x and m_z are means of x and z, respectively [35]. In this paper, x and z represent vectors of time series data with equal lengths. r is statistically significant if $p < \alpha = 0.05$, otherwise the correlation is insignificant.

Appendix 6: Vector autoregressive (VAR) models

The general VAR model of n dependent variables with independent variable t and l lags, is given by

$$v_t = \mu + \sum_{i=1}^{l} \gamma_i v_{t-i} + \varepsilon_t, \qquad (5)$$

where v_t is an $n \times 1$ vector representing time series variables at time t; μ is an $n \times 1$ vector of constants (intercepts with the v_t-axis) of time series; γ_i (i = 1, 2, ..., l) is an $n \times n$ matrix of coefficients of lagged variables at time $t - i$; ε_t is an $n \times 1$ vector of error terms (white noise) [32, 33, 34, 35].

VAR models are used to provide estimates of stationary time series, i.e. those with no time-trends in the mean and variance of the data. Our time series were detrended by differencing to achieve stationarity (confirmed by Augmented Dickey-Fuller (ADF) tests). Steps for fitting VAR models to time series data included selection of suitable lag lengths for model variables; estimation of model parameters and coefficients; testing of time series Granger-causality; and forecast error variance decomposition (FEVD) of variables.

Granger-causality estimates the degree to which one time series predicts future values of another time series. The statistical test for Granger-causality compares the predictive ability of the time series model of interest with and without the putative causal variable and is therefore interpretable as evidence towards a causal relationship, but not as proof of a causal relationship. FEVD is a statistical tool for measuring the amount of variation in a variable over time that is attributable to the variable's own past values versus past values of other model variables. Based on a fitted VAR model, FEVD plots illustrate variation over time prospectively, i.e., as a forecast. We present both FEVD and Granger-causality tests for our time series models in order to assess to what degree, and over what time frame, contact rates may be driving changes in

COVID-19 transmission and new cases.

Appendix 6.1: Stationarization of time series for average daily contacts, new cases and *Rt* in Figure 1

VAR models provide feasible estimates of stationary time series (stationary processes). A stationary process has no time-trends in the mean and variance of the data. Clearly, all time series in Figure 1 are not stationary. Before fitting VAR models, we first stationarize (detrend) the time series by differencing. Differencing works by transforming time series data such that values of the new time series are differences between consecutive values of the original time series. If the resulting time series is not stationary, then differencing can be applied consecutively more than once leading to the second-order difference, third-order difference, etc. We ascertained stationarity of our time series by applying Augmented Dickey-Fuller (ADF) test. According to ADF test, a time series is stationary if $p < 0.05$. ADF test exists in the *tseries* package in R. Stationary processes of all time series in Figure 1 are presented in Figure 3. Second-order differencing was applied to stationarize time series for average daily contacts, new cases and R_t in BC and VCHA, while third-order differencing was used to stationarize time series for FHA contacts, cases and transmission.

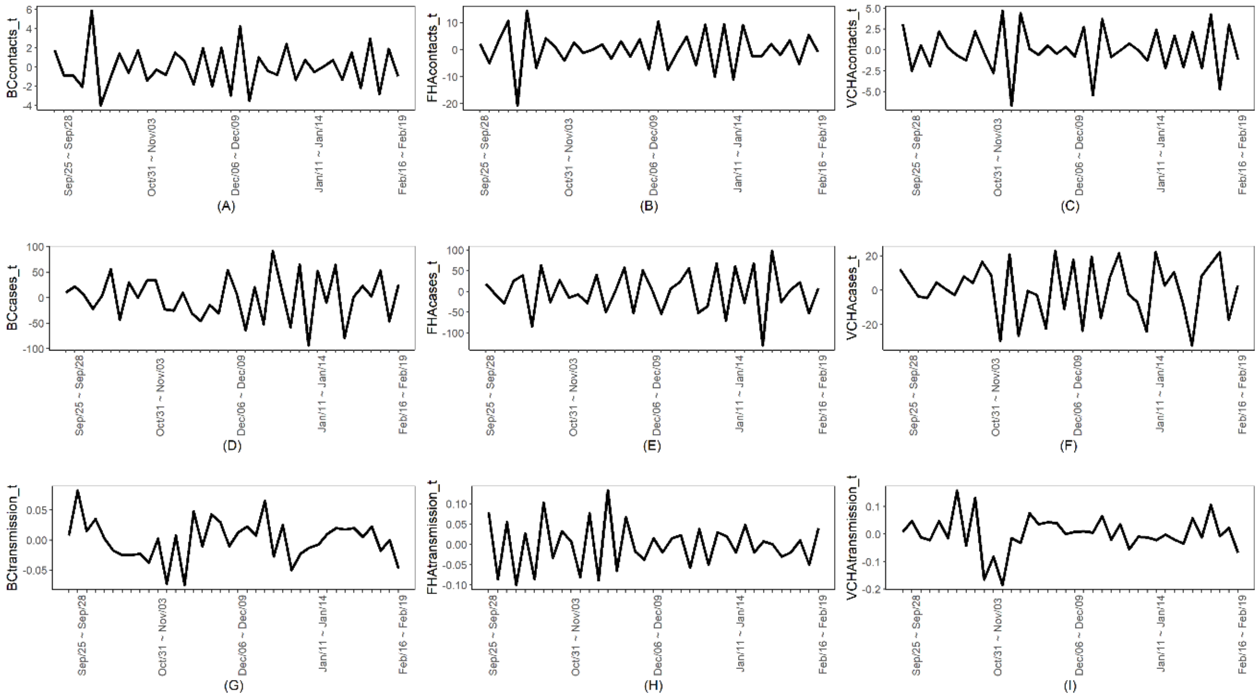

Figure 3: Stationary time series of average daily contacts, average daily cases and R_t in BC (**A, D, G**), FHA (**B, E, H**) and VCHA (**C, F, I**).

Appendix 6.2: VAR model fitting process

We applied the following steps, via the *vars* package in *R*, to fit VAR models of stationary processes of average daily contacts and new cases, and average daily contacts and R_t:

(i) Use of statistical information criteria (SIC) to determine optimal lag lengths of dependent variables that provide suitable model estimates. The optimal lag lengths are provided by four statistical information criteria, Akaike information criterion (AIC), Hannan Quinn (HQ), Schwarz criterion (SC) and (FPE). In this paper we adopted the lowest suggested lag lengths to derive our VAR model equations.

(ii) Estimation of model estimates μ and γ_i (see Equation (5)) for each VAR model.

(iii) Application of Granger causality test, which is used to determine whether one time series is useful in predicting another. A variable *v* is said to Granger-cause a variable *w* if $p < 0.05$, otherwise, *v* does not Granger-cause *w*.

(iv) Forecast error variance decomposition (FEVD) of model variables. In a FEVD, forecast errors are considered for each equation in the fitted VAR model, then the fitted VAR model is used to determine how much of each error estimate is coming from forecast errors in the other variable. FEVD indicates the amount of information that a variable contributes to step forecast error variance of another variable in the model.

Appendix 6.3: VAR model equations

Here, we derive equations for VAR models for stationary time series of average daily contacts and new cases, and average daily contacts and transmission of COVID-19, in BC, FHA and VCHA, based on the lowest optimal lag lengths suggested by SIC. The lowest suggested lag lengths for our VAR models are presented in **Table 6**.

Table 6: Selected lag lengths of VAR models for stationary time series of average daily contacts and new cases, and average daily contacts and new cases in BC, FHA and VCHA.

VAR model variables	Lag length	SIC
$BC_{contacts\_t}$ vs. BC_{cases_t}	1	AIC, HQ, SC, FPE
$BC_{contacts\_t}$ vs. $BC_{transmission\_t}$	4	SC
$FHA_{contacts\_t}$ vs. FHA_{cases_t}	6	FPE
$FHA_{contacts\_t}$ vs. $FHA_{transmission\_t}$	9	FPE
$VCHA_{contacts\_t}$ vs. $VCHA_{cases\_t}$	2	SC
$VCHA_{contacts\_t}$ vs. $VCHA_{transmission\_t}$	10	AIC, HQ, SC, FPE

Therefore, according to Equation (5), the VAR(1) model of $BC_{contacts\_t}$ and BC_{cases_t} is given by

$$BC_{contacts\_t} = \mu_1 + \gamma_{11}^1 BC_{contacts\_t-1} + \gamma_{12}^1 BC_{cases\_t-1}$$
$$BC_{cases\_t} = \mu_2 + \gamma_{21}^1 BC_{contacts\_t-1} + \gamma_{22}^1 BC_{cases\_t-1};$$

where γ_{jk}^i ($i, j, k = 1, 2$) is a matrix of coefficients of lagged variables at time $t - i$. Similarly, the VAR(4) model of $BC_{contacts\_t}$ and $BC_{transmission\_t}$ is given by

$$BC_{contacts\_t} = \mu_1 + \gamma_{11}^1 BC_{contacts\_t-1} + \gamma_{12}^1 BC_{transmission\_t-1} + \cdots + \gamma_{11}^4 BC_{contacts\_t-4} + \gamma_{12}^4 BC_{contacts\_t-4}$$

$$BC_{transmission\_t} = \mu_2 + \gamma_{21}^1 BC_{contacts\_t-1} + \gamma_{22}^1 BC_{transmission\_t-1} + \cdots + \gamma_{21}^4 BC_{contacts\_t-4} + \gamma_{22}^4 BC_{contacts\_t-4}.$$

The VAR models for $FHA_{contacts\_t}$ and FHA_{cases_t}, $FHA_{contacts\_t}$ and $FHA_{transmission\_t}$, $VCHA_{contacts\_t}$ and $VCHA_{cases\_t}$, and $VCHA_{contacts\_t}$ and $VCHA_{transmission\_t}$ were derived similarly.

Appendix 6.4: Estimates for VAR models of average daily contacts, new cases and *Rt*

The tables below show estimated values of coefficients γ_{jk}^i ($i, j, k = 1, 2..., l$), where l is the lag length, for our VAR models of contact rates and cases, and contact rates and transmission, in BC, FHA and VCHA.

Table 7: Estimation results for VAR(1) model of variables $BC_{contacts\_t}$ and BC_{cases_t}

	Dependent variable:	
	$BC_{contacts\_t}$	BC_{cases_t}
$BC_{contacts\_t-1}$	– 0.770***	7.930***
	(0.112)	(2.804)
BC_{cases_t-1}	—0.011*	– 0.400***
	(0.005)	(0.137)
Observations	37	37
R^2	0.577	0.375
Adjusted R^2	0.553	0.339
Residual Std. Error (df = 35)	1.399	35.071
F Statistic (df = 2; 35)	23.850***	10.504***
Note:	*p<0.1; **p<0.05; ***p<0.01	

Table 8: Estimation results for VAR(4) model of variables $BC_{contacts\_t}$ and $BC_{tranmission\_t}$

	Dependent variable:	
	$BC_{contacts\_t}$	$BC_{tranmission\_t}$
$BC_{contacts\_t-1}$	−1.482***	0.001
	(0.144)	(0.004)
$BC_{transmission\_t-1}$	7.907	−0.018
	(6.886)	(0.171)
$BC_{contacts\_t-2}$	−1.283***	0.003
	(0.215)	(0.005)
$BC_{transmission\_t-2}$	−5.839	0.832***
	(6.836)	(0.170)
$BC_{contacts\_t-3}$	−1.008***	0.002
	(0.213)	(0.005)
$BC_{transmission\_t-3}$	−7.574	−0.116
	(6.338)	(0.158)
$BC_{contacts\_t-4}$	−0.493***	0.002
	(0.146)	(0.004)
$BC_{transmission\_t-4}$	8.520	−0.463***
	(6.429)	(0.160)
Observations	34	34
R^2	0.837	0.528
Adjusted R^2	0.786	0.383
Residual Std. Error (df = 26)	0.990	0.025
F Statistic (df = 8; 26)	16.643***	3.639***
Note:	*p<0.1; **p<0.05; ***p<0.01	

Table 9: Estimation results for VAR(6) model of variables $FHA_{contacts\_t}$ and FHA_{cases_t}

	Dependent variable:	
	$FHA_{contacts\_t}$	FHA_{cases_t}
$FHA_{contacts\_t-1}$	−1.937***	−1.134
	(0.152)	(3.630)
FHA_{cases_t-1}	0.004	−1.182***
	(0.009)	(0.223)
$FHA_{contacts\_t-2}$	−2.051***	−5.295
	(0.289)	(6.906)
FHA_{cases_t-2}	0.024*	−1.355***
	(0.014)	(0.328)
$FHA_{contacts\_t-3}$	−1.886***	−5.200
	(0.332)	(7.931)
FHA_{cases_t-3}	0.021	−1.128**
	(0.017)	(0.415)
$FHA_{contacts\_t-4}$	−1.497***	−6.283
	(0.306)	(7.301)
FHA_{cases_t-4}	0.022	−0.945**
	(0.017)	(0.406)
$FHA_{contacts\_t-5}$	−0.925***	−4.017
	(0.220)	(5.254)
FHA_{cases_t-5}	0.018	−0.608
	(0.015)	(0.367)
$FHA_{contacts\_t-6}$	−0.407***	−2.137
	(0.107)	(2.559)
FHA_{cases_t-6}	−0.017	−0.173
	(0.010)	(0.249)
Observations	31	31
R^2	0.969	0.779
Adjusted R^2	0.949	0.639
Residual Std. Error (df = 19)	1.267	30.241
F Statistic (df = 12; 19)	48.749***	5.571***
Note:	*p<0.1; **p<0.05; ***p<0.01	

Table 10: Estimation results for VAR(9) model of variables $FHA_{contacts\_t}$ and $FHA_{transmission\_t}$

	Dependent variable:	
	$FHA_{contacts\_t}$	$FHA_{transmission\_t}$
$FHA_{contacts\_t-1}$	−2.383***	−0.005
	(0.359)	(0.004)
$FHA_{transmission\_t-1}$	25.423	−0.205
	(28.455)	(0.351)
$FHA_{contacts\_t-2}$	−3.262***	−0.011
	(0.863)	(0.011)
$FHA_{transmission\_t-2}$	8.624	0.926**
	(25.930)	(0.320)
$FHA_{contacts\_t-3}$	−3.387**	−0.014
	(1.320)	(0.016)
$FHA_{transmission\_t-3}$	−45.134	−0.046
	(28.947)	(0.357)
$FHA_{contacts\_t-4}$	−2.960*	−0.013
	(1.571)	(0.019)
$FHA_{transmission\_t-4}$	−29.659	−1.155***
	(25.977)	(0.320)
$FHA_{contacts\_t-5}$	−2.470	−0.010
	(1.575)	(0.019)
$FHA_{transmission\_t-5}$	40.356	0.050
	(38.008)	(0.469)
$FHA_{contacts\_t-6}$	−1.709	−0.006
	(1.385)	(0.017)
$FHA_{transmission\_t-6}$	7.670	0.468
	(22.508)	(0.277)
$FHA_{contacts\_t-7}$	−0.717	−0.005
	(1.060)	(0.013)
$FHA_{transmission\_t-7}$	−53.504*	−0.314
	(26.092)	(0.322)
$FHA_{contacts\_t-8}$	−0.209	−0.002
	(0.621)	(0.008)
$FHA_{transmission\_t-8}$	−22.702	−0.469
	(25.536)	(0.315)
$FHA_{contacts\_t-9}$	−0.095	−0.002
	(0.274)	(0.003)
$FHA_{transmission\_t-9}$	17.819	−0.163
	(21.252)	(0.262)
Observations	28	28
R^2	0.962	0.922
Adjusted R^2	0.894	0.780
Residual Std. Error (df = 10)	1.855	0.023
F Statistic (df = 18; 10)	14.079***	6.522***

Table 11: Estimation results for VAR(2) model of variables $VCHA_{contacts\_t}$ and $VCHA_{cases\_t}$

	Dependent variable:	
	$VCHA_{contacts\_t}$	$VCHA_{cases\_t}$
$VCHA_{contacts\_t-1}$	– 1.194***	4.421***
	(0.152)	(1.461)
$VCHA_{cases\_t-1}$	0.008	– 0.268
	(0.018)	(0.172)
$VCHA_{contacts\_t-2}$	– 0.584***	2.553*
	(0.157)	(1.506)
$VCHA_{cases\_t-2}$	—0.015	–0.074
	(0.017)	(0.164)
Observations	36	36
R^2	0.741	0.354
Adjusted R^2	0.708	0.274
Residual Std. Error (df = 32)	1.406	13.526
F Statistic (df = 4; 32)	22.836***	4.393***
Note:	*p<0.1; **p<0.05; ***p<0.01	

Table 12: Estimation results for VAR(10) model of variables $VCHA_{contacts\_t}$ and $VCHA_{transmission\_t}$

	Dependent variable:	
	$VCHA_{contacts\_t}$	$VCHA_{transmission\_t}$
$VCHA_{contacts\_t-1}$	−1.395***	0.010
	(0.274)	(0.010)
$VCHA_{transmission\_t-1}$	5.984	0.284
	(7.293)	(0.258)
$VCHA_{contacts\_t-2}$	−1.090*	0.007
	(0.507)	(0.018)
$VCHA_{transmission\_t-2}$	−5.706	0.589**
	(6.215)	(0.220)
$VCHA_{contacts\_t-3}$	−0.706	−0.007
	(0.565)	(0.020)
$VCHA_{transmission\_t-3}$	10.938	−0.508
	(8.920)	(0.316)
$VCHA_{contacts\_t-4}$	−0.643	−0.002
	(0.555)	(0.020)
$VCHA_{transmission\_t-4}$	−0.152	−0.673*
	(9.099)	(0.322)
$VCHA_{contacts\_t-5}$	−0.693	−0.007
	(0.577)	(0.020)
$VCHA_{transmission\_t-5}$	−12.496	0.503
	(10.769)	(0.381)
$VCHA_{contacts\_t-6}$	−0.244	0.004
	(0.601)	(0.021)
$VCHA_{transmission\_t-6}$	−1.131	0.186
	(10.323)	(0.366)
$VCHA_{contacts\_t-7}$	−0.560	0.015
	(0.542)	(0.019)
$VCHA_{transmission\_t-7}$	14.575	−0.664*
	(9.494)	(0.336)
$VCHA_{contacts\_t-8}$	−0.511	0.026
	(0.544)	(0.019)
$VCHA_{transmission\_t-8}$	−1.761	0.167
	(9.496)	(0.336)
$VCHA_{contacts\_t-9}$	−0.200	0.020
	(0.472)	(0.017)
$VCHA_{transmission\_t-9}$	−15.999**	−0.127
	(5.955)	(0.211)
$VCHA_{contacts\_t-10}$	0.085	0.005
	(0.240)	(0.009)
$VCHA_{transmission\_t-10}$	13.138*	−0.137
	(6.293)	(0.223)
Observations	28	28
R^2	0.982	0.938
Adjusted R^2	0.936	0.781
Residual Std. Error (df = 8)	0.721	0.026
F Statistic (df = 20; 8)	21.482***	6.006***

Appendix 6.5: Numerical representations of forecast error variance decomposition (FEVD) results in Figure 2 and illustration of Granger causality results shown in Table 2.

Table 13: Numerical representations of forecast error variance decomposition (FEVD) results (in Figure 2) for VAR models of average daily contacts and cases, and average daily contacts and transmission in BC **(A,B),** FHA **(C,D)**, and VCHA **(E,F)**.

(A) MODEL: contacts vs. cases in BC

	FEVD for BC contacts		FEVD for BC cases	
Horizon	BCcontacts_t	BCcases_t	BCcontacts_t	BCcases_t
1	1.00	0.00	0.00	1.00
2	0.96	0.04	0.07	0.93
3	0.91	0.09	0.16	0.84
4	0.89	0.11	0.22	0.78
5	0.88	0.12	0.24	0.76
6	0.87	0.13	0.25	0.75
7	0.87	0.13	0.25	0.75
8	0.87	0.13	0.25	0.75
9	0.87	0.13	0.25	0.75
10	0.87	0.13	0.25	0.75

(B) MODEL: contacts vs. transmission in BC

	FEVD for BC contacts		FEVD for BC transmission	
Horizon	BCcontacts_t	BCtransmission_t	BCcontacts_t	BCtransmission_t
1	1.00	0.00	0.16	0.84
2	0.99	0.01	0.16	0.84
3	0.95	0.05	0.19	0.81
4	0.92	0.08	0.19	0.81
5	0.92	0.08	0.20	0.80
6	0.92	0.08	0.22	0.78
7	0.91	0.09	0.21	0.79
8	0.89	0.11	0.23	0.77
9	0.85	0.15	0.22	0.78
10	0.82	0.18	0.22	0.78

(C) MODEL: contacts vs. cases in FHA

	FEVD for FHA contacts		FEVD for FHA cases	
Horizon	FHAcontacts_	FHAcases_t	FHAcontacts_t	FHAcases_t
1	1.00	0.00	0.14	0.86
2	1.00	0.00	0.13	0.87
3	0.99	0.01	0.13	0.87
4	0.92	0.08	0.13	0.87
5	0.85	0.15	0.15	0.85
6	0.81	0.19	0.17	0.83
7	0.81	0.19	0.20	0.80
8	0.65	0.35	0.21	0.79
9	0.49	0.51	0.22	0.78
10	0.45	0.55	0.22	0.78

(D) MODEL: contacts vs. transmission in FHA

	FEVD for FHA contacts		FEVD for FHA transmission	
Horizon	FHAcontacts_t	FHAtransmission_t	FHAcontacts_t	FHAtransmission_t
1	1.00	0.00	0.34	0.66
2	0.99	0.01	0.49	0.51
3	0.96	0.04	0.51	0.49
4	0.96	0.04	0.59	0.41
5	0.95	0.05	0.61	0.39
6	0.95	0.05	0.61	0.39
7	0.94	0.06	0.56	0.44
8	0.93	0.07	0.55	0.45
9	0.93	0.07	0.52	0.48
10	0.93	0.07	0.53	0.47

(E) MODEL: contacts vs. cases in VCHA

	FEVD for VCHA contacts		FEVD for VCHA cases	
Horizon	VCHAcontact	VCHAcases_t	VCHAcontacts_t	VCHAcases_t
1	1.00	0.00	0.00	1.00
2	1.00	0.00	0.18	0.82
3	0.98	0.02	0.27	0.73
4	0.95	0.05	0.28	0.72
5	0.94	0.06	0.28	0.72
6	0.94	0.06	0.29	0.71
7	0.94	0.06	0.29	0.71
8	0.94	0.06	0.30	0.70
9	0.94	0.06	0.30	0.70
10	0.94	0.06	0.30	0.70

(F) MODEL: contacts vs. transmission in VCHA

	FEVD for VCHA contacts		FEVD for VCHA transmission	
Horizon	VCHAcontacts_	VCHAtransmission_t	VCHAcontacts_t	VCHAtransmission_t
1	1.00	0.00	0.06	0.94
2	0.99	0.01	0.15	0.85
3	0.94	0.06	0.11	0.89
4	0.79	0.21	0.12	0.88
5	0.66	0.34	0.11	0.89
6	0.65	0.35	0.26	0.74
7	0.65	0.35	0.27	0.73
8	0.65	0.35	0.27	0.73
9	0.67	0.33	0.35	0.65
10	0.67	0.33	0.36	0.64

Figure 4. Pictorial representation of Granger causality results in Table 2.

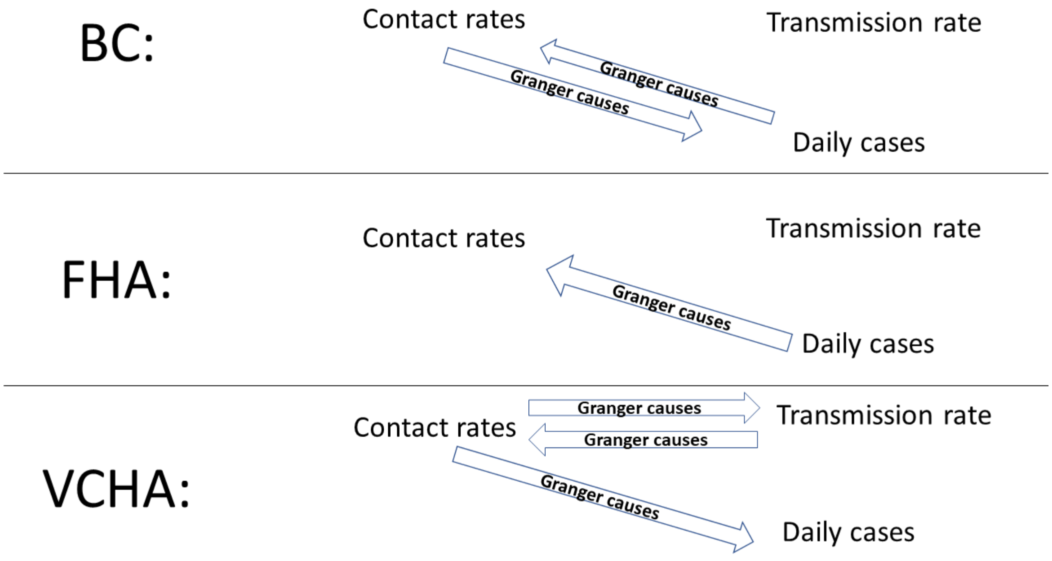

Supplement: Supplementary file 1 [file Data_Sheet_1.PDF]
